# The Graduates

**Published:** 1882-04

**Authors:** 


### THE GRADUATES.

In the May number of the REGISTER will be given a full list of the graduates of the dental colleges of the country for the terms just ended.

